# Artificially produced rare-earth free cosmic magnet

**DOI:** 10.1038/srep16627

**Published:** 2015-11-16

**Authors:** Akihiro Makino, Parmanand Sharma, Kazuhisa Sato, Akira Takeuchi, Yan Zhang, Kana Takenaka

**Affiliations:** 1 Tohoku University, Sendai 980-8577, Japan; 2 Institute for Materials Research, Tohoku University, Sendai 980-8577, Japan

## Abstract

Chemically ordered hard magnetic L1_0_-FeNi phase of higher grade than cosmic meteorites is produced artificially. Present alloy design shortens the formation time from hundreds of millions of years for natural meteorites to less than 300 hours. Electron diffraction detects four-fold 110 superlattice reflections and a high chemical order parameter (S 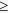 0.8) for the developed L1_0_-FeNi phase. The magnetic field of more than 3.5 kOe is required for the switching of magnetization. Experimental results along with computer simulation suggest that the ordered phase is formed due to three factors related to the amorphous state: high diffusion rates of the constituent elements at lower temperatures when crystallizing, a large driving force for precipitation of the L1_0_ phase, and the possible presence of L1_0_ clusters. Present results can resolve mineral exhaustion issues in the development of next-generation hard magnetic materials because the alloys are free from rare-earth elements, and the technique is well suited for mass production.

Iron meteorites with Widmannstaetten type of structure are mainly composed of iron and nickel. This structure is formed through an extremely slow cooling rate of about one Kelvin per million years in the universe^1^. The Widmannstaetten structure observed in Octahedrite-type meteorites is peculiar, and results from the apparent phase separation of the α-phase (*bcc*-FeNi with a mineral name: kamacite) and γ-phase (*fcc*-FeNi: taenite) at the FeNi interface. The taenite phase lamellae observed in the meteorite have varying Ni concentration zones (28–50%)^2^. Both the disordered *fcc* and ordered L1_0_ phases of Fe-Ni were detected. Interestingly, the L1_0_ FeNi phase, which is also known as tetrataenite is a hard magnetic with a high saturation magnetization (*M*_*s*_ ~ 1270 emu cm^−3^) and a large uniaxial magneto-crystalline anisotropy (*K*_*u*_ ~ 1.3 × 10^7^ erg cm^−3^)^3^,^4^,^5^. The theoretical maximum magnetic energy product of L1_0_ FeNi (~42 MG Oe) is close to the best rare-earth-based hard magnets recently developed^6^.

Due to shortage of rare-earth elements, which are currently used to produce high-grade permanent magnets, magnets free of rare-earth elements must be developed (i.e., hard magnets based on L1_0_ FeNi). Practically, it is impossible to produce L1_0_ FeNi magnet similar to meteorites industrially because the order-disorder transition temperature of L1_0_ FeNi is too low 320 °C^2^,^6^. The diffusion coefficients of Fe and Ni are extremely low around this temperature, and in reality, no diffusion takes place, which is why the ordered L1_0_ FeNi phase requires billions of years to form in cosmic products (meteorites). Since the discovery of the L1_0_ FeNi phase in the 1960 s, several attempts (which might trigger atomic migration) such as irradiation with high-energy beams^7^, a nanoparticle technique^8^, mechanical alloying^9^, thin films comprised of mono-layered atoms^10^, and high-pressure torsion technique^11^ have been tried to artificially produce this phase. However, L1_0_ FeNi-based hard magnets with high degree of chemical order have yet to be produced.

It seems that the production of L1_0_ FeNi-based hard magnets via conventional material synthesis utilizing atomic diffusion in the crystalline state is extremely difficult, if not impossible. The high stability of crystalline phase and the extremely low diffusivity of the atoms around the order-disorder transition temperature (~320 °C) in crystalline alloys are the biggest hurdles. A high diffusivity of atoms at lower temperatures may successfully form the L1_0_ FeNi phase, but it is difficult to achieve. Although high-pressure torsion and high-energy ball-milling techniques can increase the diffusivity of atoms^11^, the increase is insufficient. A high atomic diffusivity is possible at low temperatures, such as the transformation from an amorphous state to a stable crystalline state. Distribution of elements in the amorphous alloy is similar to the initial state of meteorites (chondrule). At the dawn of the universe, chondrules were formed by the condensation of solar nebula and melting of condensed mineral clumps. A liquid like state in an amorphous alloy (similar to chondrule) can exist at room temperature. The major difference in approaching a stable crystalline state from the amorphous state is the drastic increase in diffusivity at the transition temperature (i.e., crystallization temperature), whereas cooling molten alloys decreases the diffusivity at the transition temperature. If an alloy based on ~Fe_50_Ni_50_ can be made into an amorphous state with a crystallization temperature close to the order-disorder transition temperature, then L1_0_ FeNi-based magnets may be realized.

## Results

Various Fe-based amorphous alloys have been developed^12^. Generally, the crystallization temperature of these alloys is much higher (>450 °C) than the order-disorder transition temperature of the L1_0_ FeNi phase. Recently, we have developed new high magnetic flux density FeSiBPCu-based nanocrystalline soft magnetic alloys^13^,^14^. The initial state of the FeSiBPCu alloy is amorphous, but it crystallizes into nano-crystalline α-Fe in the remaining amorphous matrix below 400 °C. Crystallization of this amorphous alloy is very rapid (i.e., the atomic diffusivity of the constituent elements is very high)^15^. Additionally this alloy also contains Phosphorus (P), which is also present in the NWA 6259 meteorite^6^. These characteristics are very promising for developing L1_0_ FeNi-based hard magnets.

Therefore, we replaced Ni with Fe in the FeSiBPCu alloy. The results suggest that the Fe_42_Ni_41.3_Si_8_B_4_P_4_Cu_0.7_ alloy is the best among the investigated compositions. It should be noted that we also investigated crystalline FeNi binary alloys, but the formation of L1_0_ FeNi could not be detected. In Fe_42_Ni_41.3_Si_x_B_12–x_P_4_Cu_0.7_ (x = 2 to 8 at.%) alloys, a higher Si content is better for the formation of the L1_0_ FeNi phase.

**Figure 1:**
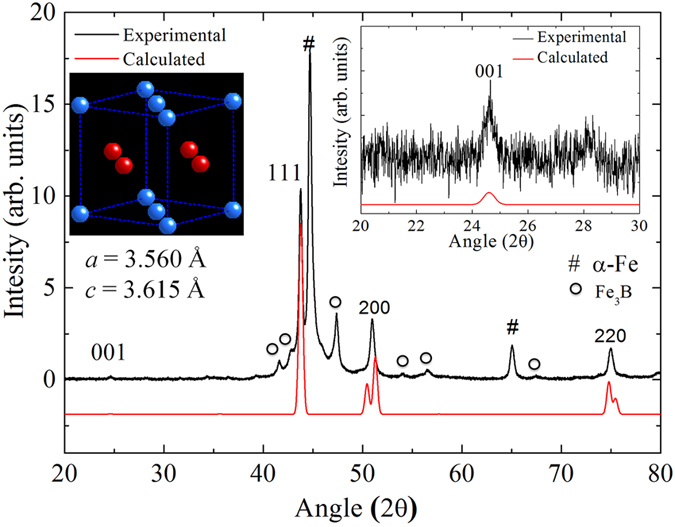
Experimental (black) and calculated X-ray diffraction patterns (red) of the L1_0_ phase. Right inset is a magnified graph at 2θ ranging from 20 to 30 degrees for the (001) super-lattice diffraction. Left inset demonstrates the atomic arrangements of the L1_0_ phase with Fe (blue) and Ni (red) atoms drawn with lattice parameters of *a* = 3.560 and *c* = 3.615 Å.

The as-quenched state of the Fe_42_Ni_41.3_Si_8_B_4_P_4_Cu_0.7_ alloy is amorphous and its crystallization temperature measured by differential scanning calorimetry (DSC) is ~400 °C (at a heating rate of 40 °C/minute). Figure 1 shows the X-ray diffraction (XRD) pattern of the Fe_42_Ni_41.3_Si_8_B_4_P_4_Cu_0.7_ ribbon crystallized at 400 °C for 288 hours. The diffraction peaks corresponding to the ordered L1_0_ FeNi phase (inset of Fig. 1) along with α-Fe and Fe_3_B phases are also detected.

The experimental X-ray diffraction pattern was fit to the L1_0_ FeNi phase. The lattice constants, *a* and *c*, were evaluated to be 0.3560 and 0.3615 nm, respectively, which agree well with a natural meteorite (*a* = 0.3582 nm and *c* = 0.3607 nm)^16^. The intensity of the superlattice reflections is weak because Fe and Ni have similar atomic scattering factors. The calculated intensity of the (001) superlattice reflection is 0.3% of the (111) fundamental reflection, which is much lower than the experimentally observed value (~1.7%), suggesting that the ribbon has a texture in the out-of-plane direction i.e. c-axis.

**Figure 2:**
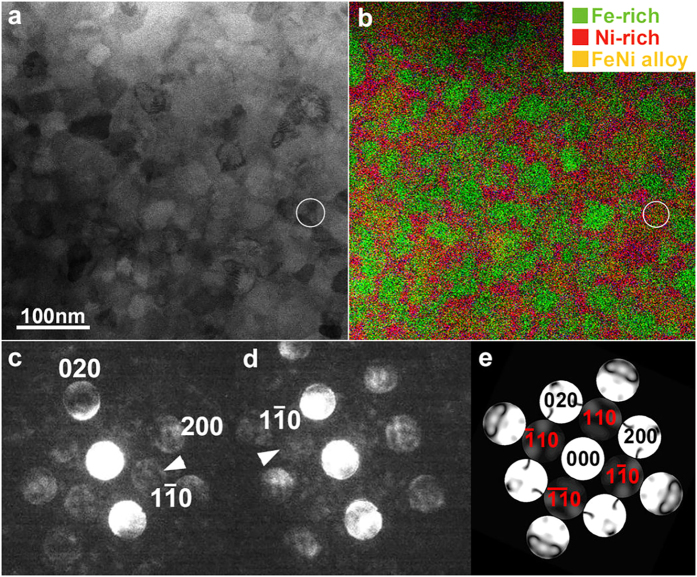
Observations by electron microscopy. (**a**) STEM-bright field image and (**b**) STEM-EDX elemental mapping. (**c**,**d**) Nanobeam electron diffraction taken from the area marked with circles in Fig. (a,b). (**e**) Simulated NBD pattern of the L1_0_ FeNi structure with *S* = 0.8.

Figure 2a shows a bright-field (BF) scanning transmission electron microscope (STEM) image of the Fe_42_Ni_41.3_Si_8_B_4_P_4_Cu_0.7_ alloy after annealing at 400 °C for 288 hours. The microstructure is composed of 30-50 nm sized polycrystalline grains. Elemental mapping by energy dispersive X-ray spectroscopy (EDX) using STEM reveals that these grains include at least three phases: an Fe-rich phase, a Ni-rich phase and a nearly equi-atomic Fe_50_Ni_50_ alloy phase (Fig. 2b). It should be mentioned that Si and P were detected in the Ni-rich grains, but not in the Fe-rich or FeNi grains. Detection of Fe_3_B phase by XRD indicates that B is distributed in Fe-rich phase. Areal fraction of these three phases are 40% (Ni-rich), 37% (Fe-rich), and 23% (Fe-Ni alloy). Thus, partitioning of the solute elements indicates that the Fe-rich grains correspond to the α-Fe and Fe_3_B phases as detected by the X-ray measurements (Fig. 1). The Ni-rich grains are *fcc*, and the equi-atomic Fe_50_Ni_50_ regions are possibly made from L1_0_ or *fcc* type of grains.

We obtained the nanobeam electron diffraction (NBD) patterns (probe size ~0.5 nm), which include, the superlattice reflections from an area within the equi-atomic FeNi alloy phase. Figure 2c,d show the [001]-zone NBD patterns obtained by scanning a grain marked by an encircled region in Fig. 2a,b. The existence of four-fold 110 superlattice reflections clearly indicates that the L1_0_-type ordered phase is formed with the *c*-axis oriented normal to the specimen plane. This is consistent with the XRD measurements. Volume fraction of the L1_0_ phase is roughly estimated to be ~8% based on the STEM-EDX elemental map together with NBD patterns by assuming a spatially random orientation of the c-axis. The long-range order (LRO) parameter (*S*) is approximately ~0.8 or above, which was estimated based on simulations of the NBD patterns as a function of LRO parameters. Surprisingly, this value is higher than that reported for natural meteorites (*S* = 0.608)^16^. It is possible that *S* is reduced due to heating of a natural meteorite surface upon entering the Earth’s atmosphere.

Figure 2e shows the simulated NBD pattern of the L1_0_-FeNi structure with *S* = 0.8. Superlattice reflections are marked in red color. It should be mentioned that the polycrystalline nature may prevent frequent detection of the ordered structure; intensity of the superlattice reflection is sensitively degraded by misorientation from the exact zone axis. Additionally, the possible distribution of the degree of order cannot be detected; we found that the superlattice reflections are too weak, and practically invisible in simulations when *S* is below 0.75. Regardless of the aforementioned effects, we have successfully detected the single crystal electron diffraction patterns as demonstrated in Fig. 2c,d. This is a strong experimental evidence for the L1_0_ phase formation. Thus, the state-of-the-art electron imaging unveils the presence of the highly ordered L1_0_ phase in annealed Fe_42_Ni_41.3_Si_8_B_4_P_4_Cu_0.7_ ribbons. Here, it is worth mentioning that such a high degree of chemical order and a clear presence of superlattice reflection for L1_0_ FeNi have not been reported yet.

**Figure 3:**
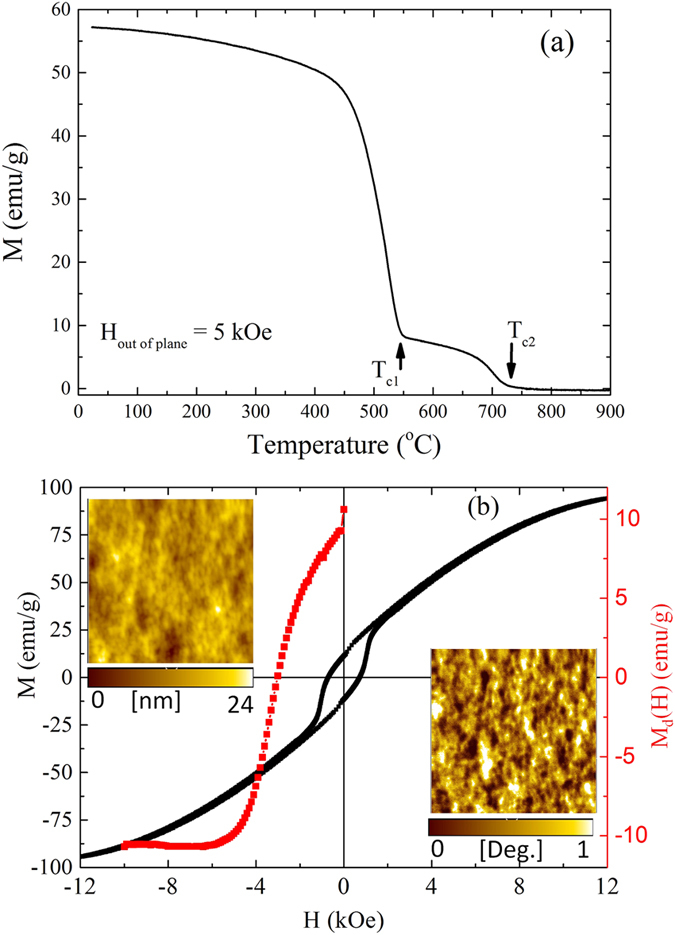
Magnetic properties of Fe_42_Ni_41.3_Si_8_B_4_P_4_Cu_0.7_ alloy. (**a**) Temperature dependent magnetization curve measured by applying a magnetic field of 5 kOe in out of plane, (**b**) Magnetic hysteresis (left: black) and dc demagnetization curves (right: red). Measurement conditions include applying a maximum magnetic field of ~12 kOe perpendicular to the ribbon plane for M Vs H curve and 10 kOe for Md(H) Vs H curve. Top inset shows the topographic AFM image, while the bottom inset is the corresponding magnetic force microscopy (MFM) image demonstrating the magnetic domains.

Figure 3a shows the temperature dependent magnetization curve for the Fe_42_Ni_41.3_Si_8_B_4_P_4_Cu_0.7_ alloy after annealing at 400 °C for 288 hours. There are two magnetic phases with Curie temperatures T_c1_ ~ 500-550 °C, and T_c2_ ~ 700-750 °C. Based on our structural analysis and reported literature^17^,^18^, the T_c1_ could be for L1_0_ FeNi or Fe_3_B, and T_c2_ for α-Fe phases. Metalloid rich phases are believed to be non-magnetic at/above room temperature. Among all the magnetic phases present in the alloy, only L1_0_ FeNi is hard magnetic. If it is so, the hysteresis curve can reveal the presence of soft and hard magnetic phases. Figure 3b shows the hysteresis curve measured by applying a maximum magnetic field of ~12,000 Oe perpendicular to the ribbon plane. The saturation magnetization (*M*_*s*_) and coercivity are ~100 emu/g and 700 Oe, respectively. A rapid increase in the magnetization at a lower magnetic field and the linear variation at higher magnetic fields suggest two processes: (1) alignment of the out-of-plane magnetization at low fields by the domain wall motion and (2) rotation of the in-plane magnetization to the out-of-plane at higher fields. The second process can be easily understood based on the presence of soft magnetic phases (α Fe and Fe_3_B phases), which have magnetic easy axes in the ribbon plane.

The magnetic easy axis of L1_0_ FeNi is along the c-axis, which is perpendicular to the ribbon plane (due to texture). It seems the alignment of the out-of-plane magnetization at a lower field by the domain wall motion is due to the presence of hard magnetic L1_0_ FeNi grains whose c-axis is along the field direction. Since the sample is polycrystalline, grains with mutually orthogonal c-axis ie. along [100] and [010] can contribute to linear increase in magnetization at higher fields (in addition to soft magnetic α-Fe and Fe_3_B phases). Such a linear increase in magnetization was also observed for the meteorites^3^,^6^. In the absence of a magnetic field, the magnetization of grains tends to remain along the easy axis of magnetization (i.e., out-of-plane for L1_0_ FeNi and in-plane for the soft magnetic phases). Therefore, the remanent magnetization (*M*_*r*_) in Fig. 3b is approximately due the L1_0_ FeNi grains, but the *H*_c_ in the out-of-plane direction is strongly influenced by the rotation of the in-plane magnetization (higher the volume fraction of soft phase lower is the *H*_*c*_)^19^. Low *M*_*r*_ (~10% of *M*_*s*_) seems to be consistent with EDX elemental mapping, which suggest volume fraction of L1_0_ phase is ~8%.

The magnetic reversal of L1_0_ FeNi can be understood from the dc demagnetization remanent [*M*_*d*_*(H*)] curve (Fig. 3b). Basically, *M*_*d*_*(H)* is the remanent magnetization of the initially saturated L1_0_ FeNi grains upon reversing the magnetic field. Figure 3b shows that a magnetic field of at least ~3.5 kOe is required to switch the magnetization of the L1_0_ FeNi grains in the present alloy. It should be noted that the L1_0_ FeNi grains oriented in other directions, and strong demagnetizing effect resulting from high *M*_*s*_ of α-Fe grains can assist in magnetization switching at lower magnetic fields. We believe the magnetization switching field of the present L1_0_ FeNi grains is higher than 3.5 kOe, and it can increase further by increasing the volume fraction. Nevertheless, such a high switching field is consistent with the highly anisotropic nature of the L1_0_ FeNi phase.

The magnetic domains were also imaged using magnetic force microscopy (MFM). A typical MFM image along with the surface topography is shown in the inset of Fig. 3b. To eliminate surface topography effects in the MFM image, the distance between the tip and the sample surface was varied from 25 nm to 100 nm. In all the cases, MFM images show the same features, suggesting that the image contrast mainly originates from the interaction of the magnetic tip with the out-of-plane magnetization of the sample. The magnetic domain patterns of the sample (shown in the inset of Fig. 3b) are similar to other hard magnetic nano-composites with soft and hard magnetic phases^20^,^21^. Both the structural and magnetic characterizations confirm that the highly ordered L1_0_ FeNi phase is formed artificially in Fe_42_Ni_41.3_Si_8_B_4_P_4_Cu_0.7_ ribbon crystallized at 400 °C for 288 hours. Although, the annealing temperature (~400 °C) for formation of ordered phase is higher than the order-disorder transition temperature (320 °C), our measurements Fig. 3a show onset temperature for disordering is ~530 °C. The results obtained are very similar to NWA 6259 meteorite^3^,^6^.

Molecular dynamic (MD) simulations were performed to understand the phase stability (energy level). The Hamiltonians (*H*’s) for amorphous, *bcc*, *fcc*, and L1_0_ phases are –384.6, –393.3, –396.7, and –397.2 kJmol^–1^, respectively. According to *H*, the L1_0_ phase is the most stable phase, and the other phases are energetically destabilized in the sequence of *bcc*, *fcc*, and amorphous. The *G* was also calculated for the amorphous (–20.9 kJmol^–1^), *bcc* (–24.6 kJmol^–1^), and *fcc* (–29.9 kJmol^–1^) phases based on the CALPHAD method. Although we were unable to evaluate *G* for the L1_0_ phase, it is estimated to be –35 kJmol^–1^ by referring to more accurate calculations for the formation enthalpy of the L1_0_ phase based on CALPHAD^22^ and ab initio methods^23^.

**Figure 4:**
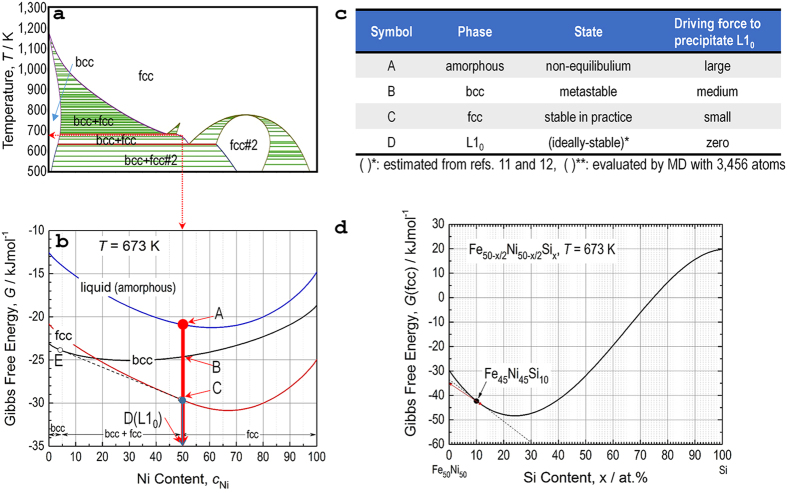
Calculated results and theoretical consideration for forming L1_0_ from the amorphous and crystalline phases. (**a**) FeNi phase diagram calculated with SSOL5 database. (**b**) Gibbs free energy curves at *T* = 673 K for liquid, *bcc*, and *fcc* phases calculated with the SSOL5 database as functions of the Ni fraction together with *G* = –35 kJmol^–1^ for the L1_0_ phase estimated from previous studies. (**c**) Characteristics of the phases of interest, including the driving force to precipitate L1_0_ at *T* = 673 K. (**d**) Changes in Gibbs free energy (*G*) of *fcc* phase as a function of Si content in a hypothetical Fe_50_Ni_50_-Fe_50−x/2_Ni_50−x/2_Si_x_ system where G = –35 kJmol^−1^ was considered for L1_0_ Fe_50_Ni_50_ phase. Based on lever rule (marked by red arrow), the volume fraction of the L1_0_ phase is evaluated to be ~13% in Fe_42_Ni_41.3_Si_8_B_4_P_4_Cu_0.7_ alloy under an assumption that L1_0_ FeNi phase precipitates from the *fcc* Fe_45_Ni_45_Si_10_, which is an equilibrium phase at *T* = 673 K in the alloy.

Figure 4 schematically diagrams the above thermodynamic results. The binary phase diagram of Fe–Ni Fig. 4a calculated using the widely accepted SSOL5 database demonstrates that Fe_50_Ni_50_ (at.%) is thermodynamically stable as a single *fcc* phase at *T* = 673 K (as drawn with both arrows). Analysis of *G* Fig. 4b also indicates a single *fcc* phase, and Fe_50_Ni_50_ is the composition at the edge of the phase separation between *bcc* (Symbol E in Fig. 4b) and *fcc* (a composition close to Symbol C marked with open circle in Fig. 4b).

Thermodynamic analysis can be used to roughly estimate the volume fraction of L1_0_ FeNi phase in Fe_42_Ni_41.3_Si_8_B_4_P_4_Cu_0.7_ alloy. Calculations were performed with Thermo-Calc by using TCFE7 database for Fe-based alloys and steels. As a result, the volume fraction of *fcc* Fe_45_Ni_45_Si_10_ phase, which is an equilibrium phase at *T* = 673 K in Fe_42_Ni_41.3_Si_8_B_4_P_4_Cu_0.7_ alloy is evaluated to be 77.6%. Probably L1_0_ FeNi phase precipitates from *fcc* Fe_45_Ni_45_Si_10_. Therefore, by assuming *G* = –35 kJmol^−1^ for L1_0_ Fe_50_Ni_50_ phase, we estimated the *G* for Fe_50_Ni_50_-Fe_50−x/2_Ni_50−x/2_Si_x_ system with different Si contents (Fig. 4d). Thermodynamically, it is possible to decompose Fe_45_Ni_45_Si_10_ into Fe_50_Ni_50_ and Fe_88_Ni_88_Si_12_. Based on lever rule (as indicated by red arrow in Fig. 4d), volume fraction of L1_0_ FeNi phase was estimated as ~1/6^th^ of 0.776 ie. ~13%. This is close to the experimental value of ~8 to 10%.

We believe that the formation of the ordered phase is due to simultaneously achieving three factors; 1. High diffusion rates of the constituent elements at lower temperatures when crystallizing from an amorphous phase, 2. A large driving force for precipitation of the L1_0_ phase from the amorphous state, and 3. Presence of compositional and structural fluctuations in the heterogeneous amorphous structure (similar to amorphous FeSiBPCu alloys^14^,^15^), which play a role of the nuclei (clusters) when forming the L1_0_ phase.

Although, the volume fraction of L1_0_ FeNi phase is low (8 ~ 13%), the hard magnetic L1_0_ phase developed in the present study is both academically and industrially novel. First, the melt-spinning technique, and low temperature annealing are able to produce the L1_0_ FeNi phase at a much faster rate than the natural process (millions of years are required for meteorites). Second, the artificial L1_0_ phase has a much higher chemical order than natural meteorites. Third, the non-equilibrium processing technique provides a new method to create a low temperature phase (such as L1_0_ FeNi), which is difficult to obtain using conventional processing. Here, it is also important to mention that the quasicrystals were first formed through non-equilibrium processing technique only, and later they were discovered even in meteorite^24^. Fourth, the present results shed light on hard magnetic materials, which have been stagnating since the discovery of rare-earth-based magnets almost 30 years ago. Fifth, thermodynamic analysis and non-equilibrium processing reported in this article should help in stimulating the research and development of new alloy systems with higher volume fractions of hard magnetic L1_0_ FeNi grains. Sixth, and most important, the realization of hard magnets free of rare-earth metals may help in resolving the global issues of resource exhaustion, which should become a critical in the near future. Hence, the successful synthesis of the chemically ordered L1_0_ FeNi phase is one-step closer to the field of materials science for realizing a safe and sustainable society in the 21^st^ century.

## Methods

### Experiments

Alloy ingots of Fe_42_Ni_41.3_Si_x_B_12–x_P_4_Cu_0.7_ (x = 2 to 8 at.%) were made by high frequency melting. These alloy ingots were used to prepare ribbons via a single roller melt-spinning technique in air. Annealing was performed by sealing the ribbons in an Ar-gas filled silica tube, which was inserted in a furnace preheated to the required annealing temperature. A Rigaku (Smart Lab) X-ray diffractometer was used to identify the structure. Commercially available software (CrystalMaker) was used to fit the experimentally obtained X-ray diffraction curves. A vibrating sample magnetometer (VSM) was used to measure the saturation magnetization (*M*_*s*_), coercivity (*H*_*c*_), and dc demagnetization remanence [*M*_*d*_*(H)*] curves. To measure *M*_*d*_*(H)* in the out-of-plane direction, the ribbon sample was exposed to a positive magnetic field of 10,000 Oe, which was applied normal to the ribbon plane. The field was made to zero and a remanence magnetization was recorded. Then a small negative magnetic field was applied and then switch to zero, and remanent magnetization was measured. Similar steps were repeated for the increasing negative magnetic field to obtain the *M*_*d*_*(H)* vs *H* curve. Some of the annealed ribbons were thinned by Ar ion milling for electron transparency. Microstructures of the specimens were characterized using a JEOL JEM-ARM200F scanning transmission electron microscope (STEM) operating at 200 kV with a CEOS aberration (Cs) corrector for the probe-forming lens and a cold field emission gun (cold-FEG). Nanobeam electron diffraction patterns were obtained by a scanning fine electron probe (probe size ~0.5 nm) with a beam convergence semi-angle of 3 mrad. Compositional analyses were carried out using an energy-dispersive X-ray spectrometer attached to the STEM. Specimen thickness was evaluated by electron energy-loss spectroscopy (EELS) in the STEM mode. The NBD patterns were simulated based on the Bloch wave calculation using the MacTempas software (Total Resolution LLC).

### Simulations and calculations

Computational methodologies include molecular dynamics (MD) simulations and calculations of the phase diagrams and Gibbs free energy (*G*) based on equilibrium thermodynamics with targets of (a) amorphous, (b) *bcc* (body-centered cubic), (c) *fcc* (face-centered cubic), and (d) L1_0_ phases using commercial software. The MD simulations were performed with Materials Explorer Version 5.0 (Fujitsu Production). For (a) amorphous, (c) *fcc*, and (d) L1_0_ phases, 4,000 atoms comprised of 2,000 Fe and 2,000 Ni atoms were dealt with in the MD simulations, which corresponds to 10 × 10 × 10 supercells in the conventional *fcc* lattice where four atoms are accommodated in the unit cell. On the other hand, 3,456 atoms in total were dealt with for (b) *bcc* phase, which corresponds to 12 × 12 × 12 supercells in the conventional *bcc* lattice with two atoms in the unit cell.

The following calculation conditions were applied to the MD simulations. The *NTp* ensemble was selected to keep the number of atoms (*N*), temperature (*T*), and pressure (*p*) constant, where *p* was set to atmospheric pressure (101.325 kPa). By an optimizing function in the software, the mass coefficient of a hypothetical heat bath in the Nosé scheme was initially determined to be 0.0643 for (a), (c), and (d) from the *fcc* supercell and 0.0529 for (b) from the *bcc* supercell. Un-distortable cubic lattices were used under GZ (GrujicicZhou)-type EAM (Embedded Atom Method) potentials with a cut-off distance of 1.02 nm (1.01 nm for (b)) and periodic boundary conditions. The aforementioned phases were created via the following procedure. (a) Amorphous phase was obtained by quenching at a cooling rate of 10^15^ K/s before and after holding the alloy for 2 ps at 1773 and 673 K, respectively. On the other hand, (b) *bcc*, (c) *fcc*, and (d) L1_0_ phases were created by holding the initial states at 673 K for 2 ps. During the common process at 673 K for 2 ps, we monitored the changes in parameters, such as *T*, *p*, and lattice constants to avoid calculation errors (e.g., overshooting). Then each phase was further annealed at 673 K for 2 ps to evaluate the physical and thermodynamic values, such as density, lattice constants, as well as *T*, *p*, and volume as the primary monitoring variables.

The thermodynamic calculations and investigations were performed based on approaches from CALPHAD (CALculation of PHAse Diagrams) and SGTE (Scientific Group Thermodata Europe)^25^. Specifically, we utilized commercial software, Thermo-Calc version 4.1 (Thermo-Calc Software AB), with the SSOL5 database for solid solutions as well as TCFE7 database for Fe-based alloys and steels equipped in the software under atmospheric pressure.

## Additional Information

**How to cite this article**: Makino, A. *et al.* Artificially produced rare-earth free cosmic magnet. *Sci. Rep.* **5**, 16627; doi: 10.1038/srep16627 (2015).
